# Improving the CONTES method for normalizing biomedical text entities with concepts from an ontology with (almost) no training data

**DOI:** 10.5808/GI.2019.17.2.e20

**Published:** 2019-06-27

**Authors:** Arnaud Ferré, Mouhamadou Ba, Robert Bossy

**Affiliations:** 1 MaIAGE, INRA, Paris-Saclay University, 78350 Jouy-en-Josas, France; 2 LIMSI, CNRS, Paris-Saclay University, 91405 Orsay, France

**Keywords:** biomedical text mining, entity normalization, ontology, word embedding

## Abstract

Entity normalization, or entity linking in the general domain, is an information extraction task that aims to annotate/bind multiple words/expressions in raw text with semantic references, such as concepts of an ontology. An ontology consists minimally of a formally organized vocabulary or hierarchy of terms, which captures knowledge of a domain. Presently, machine-learning methods, often coupled with distributional representations, achieve good performance. However, these require large training datasets, which are not always available, especially for tasks in specialized domains. CONTES (CONcept-TErm System) is a supervised method that addresses entity normalization with ontology concepts using small training datasets. CONTES has some limitations, such as it does not scale well with very large ontologies, it tends to overgeneralize predictions, and it lacks valid representations for the out-of-vocabulary words. Here, we propose to assess different methods to reduce the dimensionality in the representation of the ontology. We also propose to calibrate parameters in order to make the predictions more accurate, and to address the problem of out-of-vocabulary words, with a specific method.

**Availability:** The tool is on GitHub, https://github.com/ArnaudFerre/CONTES; and the corpus and the ontology used are available at BioNLP Shared-Task 2016, https://sites.google.com/site/bionlpst2016/tasks/bb2; pre-calculated embeddings are available on demand.

## Introduction

The world of science produces a wealth of knowledge in the form of scientific literature, a volume is so large that it is difficult or even impossible for scientists to keep abreast of new developments [1]. One solution to deal with this problem is a natural language processing approach known as information extraction [2]. Information extraction is the process of finding statements on a specific subject in texts [3]; for example, finding statements about a protein binding to another protein, or medication to treat a disease.

Normalization is the grounding of an entity mentioned in the text to an identifier in a data- or knowledge-base [4]. This is a core task of information extraction, which can be understood as a classification problem: classify entity mentions into correct class(es) from a reference vocabulary. Current normalization methods try to address two main problems: (1) the weak generalizability to different domains/tasks and (2) the high morphological variability in entity mentions and labels of classes.

Supervised approaches, combined with distributional semantics, can bring some answers. In the general domain, where the amount of training data is high, such methods already yield good results [5]. But in specialized domains, such as biomedicine, where there is often a lack of training data and/or a high number of classes, these state of the art methods seem to have difficulties to keep a high performance [6].

Recently, the CONTES (CONcept-TErm System) method [7], a supervised method, tries to address this problem by integrating more knowledge from domain ontologies. Ontologies being sometimes used as reference for normalization tasks, their hypothesis is that deeper integration could improve the quality of the predictions. The method outperforms existing approaches on the Bacteria Biotope (BB) normalization task of the BioNLP Shared Task 2016 [8]. The CONTES method, however, has two main limitations. The first is the size of the target ontology, i.e., large sets of concepts prove difficult to handle because of their high dimensionality. The second is overgeneralization: CONTES tends to “play it safe,” and predict more general concepts. Another possible limitation comes from the impossibility of the method to represent words unseen during the training step; that is, an out-of-vocabulary word problem.

Here, we propose to experiment different methods to reduce the dimensionality of the representation of the ontology structure, and to calibrate parameters to make less general predictions. We also propose to integrate another method to produce word embeddings, which addresses the problem of out-of-vocabulary words.

## Description of the CONTES Method

CONTES [7] implements a supervised machine learning approach which aims to improve the generalization limitations of previous machine learning methods, especially when few training datasets are available, with respect to the number of classes. CONTES is an open-source tool written in Python (https://github.com/ArnaudFerre/CONTES). The core of the method relies on two different semantic vector spaces: (1) semantic vector representations of expressions from a corpus (SSC for Semantic Space of Corpus), (2) semantic vector representations of concepts from an ontology (SSO for Semantic Space of Ontology).

CONTES can use all kinds of semantic vector representations for SSC computations, such as distributional representations. It particularly uses Word2Vec word embeddings [9]. For SSO computation, it automatically builds concept vectors by analyzing subsumption relationships between all the concepts of an ontology. From the vector representations, it learns a projection of the SSC into the SSO by globally maximizing the cosine similarity between the expression vectors and the associated concept vectors, in the training set. In an unsupervised setting, instead of using (expression, concept) associations, obtained from an annotated corpus for training, CONTES can use (label of concept, concept) intrinsic associations. CONTES showed great performance on the BB normalization task of BioNLP Shared Task 2016 [7]. Analysis of the results suggested that even mispredicted concepts were not far from the ontology of the expected concepts.

## Limitations

### Dimension reduction and concept embeddings

The CONTES method has been evaluated as a normalization task of BB from the BioNLP Shared Task of 2016 (http://2016.bionlp-st.org/tasks/bb2). This task consists of normalizing entity mentions, in the raw text, with concepts of the OntoBiotope ontology. OntoBiotope is an ontology of microorganism habitats, and captures classifications used by biologists to describe microorganism isolation sites (e.g., GenBank, GOLD, ATCC), and contains around 2,000 concepts, which is relatively small, compared to other biomedical ontologies, but relatively high, for a machine-learning classification problem. In comparison, the Gene Ontology contains around 50,000 concepts and the Unified Medical Language System meta-thesaurus contains more than one million concepts. CONTES does not currently address normalization with these large ontologies, due to its high dimension, but rather implies the representation of the ontology concepts.

### Overgeneralization

CONTES takes advantage of the ontology structure given by a set of *Is-A* relations between concepts. The representation of the ontology structure into SSO vectors allows CONTES to generalize training examples, and classify terms never seen before with more general concepts. However, error analysis on the BB development set showed that CONTES tends to overgeneralize its predictions, and often predicts too general concepts.

### Out-of-vocabulary words

CONTES uses the Word2Vec-CBOW and Word2Vec-SkipGram methods to produce word embeddings, and these methods do not compute valid representations for out-of-vocabulary words. Thus, CONTES does not yet address the problem of the out-of-vocabulary words. The lack of valid representations for some words is likely to bias the performance of the method. For example, in French or Spanish, most verbs have more than forty different inflected forms, while the Finnish language has fifteen cases for nouns. These languages contain many word forms that occur rarely (or not at all) in the training corpus, making it difficult to learn good word representations.

## Experiments

### Materials

For our experiments, we use the current source code of CONTES (https://github.com/ArnaudFerre/CONTES), and datasets and the OntoBiotope ontology from the BB normalization task of the BioNLP Shared Task 2016. We also downscaled word embeddings computed from a reduced corpus (40,000 words), in order to speed up the experimental cycle.

### Dimensionality-reduction techniques

We aimed to reduce the dimension of the SSO to make CONTES scalable to larger ontologies. We have used three well-known techniques to reduce the dimension of the semantic space of the ontology: principal component analysis (PCA), multidimensional scaling (MDS), and t-distributed Stochastic Neighbor Embedding (t-SNE). PCA and MDS have already been tested, with similar results [7]. Here, t-SNE is a nonlinear method to reduce dimensions, with the aim to retain the similarity of vectors between the initial space and the embedded space. All these methods can be used with the machine learning Python library scikit-learn (https://scikit-learn.org/stable/).

### Concept embeddings

Another solution to overcome the problem of high-dimensional vectors of ontology concepts, in CONTES, is to directly learn embeddings for each concept. With the success of the word-embedding methods, we considered graph-based embeddings, and used Node2Vec [10] to compute the concept vectors (SSO) of ontologies for CONTES.

Node2Vec is a method to learn embeddings for nodes in networks. The approach relies on a maximization of the likelihood of preserving neighborhoods of nodes in networks. It considers the hypothesis that highly connected nodes of the same community have similar embeddings, and that nodes having similar structural roles in networks, have similar embeddings [10].

For CONTES, Node2Vec has the advantage of being applicable to ontologies, and it also directly provides low-dimensional vectors. Although nodes in ontologies are not generally highly connected, as can be the social network graphs for example, we expect that Node2Vec can also be suitable for ontologies by providing low-dimensional vectors for ontology concepts, based on the neighborhoods expressed in relationships such as “*Is-A*” and “*sameAs*.”

The experiments with Node2vec allowed us to obtain compact vector representations for the Ontobiotope concepts, which opens the possibility for larger ontologies.

### Overgeneralization

Our hypothesis was that CONTES overgeneralization is caused by giving too much weight to the individual concept ancestors, in the vectors representing the ontology structure. CONTES uses the “Ancestry” method where each concept is represented as a vector having a number of dimensions equal to the total number of concepts in the ontology. For a given concept, the dimension corresponding to the concept itself or to one of its ancestors is set to one (1). All other dimensions are set to zero (0). The representation has the advantage of preserving the required information to rebuild the ontology structure. However, all ancestors have the same weight regardless of the number of intermediate concepts.

We experimented with a method where the dimension value is decreased using a function that depends on (1) the depth between the concept and the ancestor, and (2) a constant parameter used to set a “decay” factor. The function is w^d^, where *w* is the decay factor whose range is *[0,1]*, and *d* is the number of intermediate concepts between the target concept and its ancestor (zero for itself, one for its direct parent).

### Out-of-vocabulary words

New words currently required to recalculate the word embeddings in CONTES. Methods like FastText are designed to obtain embeddings for words even if they are unknown from the training data. For our experiments, we considered FastText [11] as a solution to tackle the problem of out-of-vocabulary words.

The FastText method is based on the skip-gram model [9,11], and takes into account subword information to produce word embeddings. It also considers each word as a bag of character n-grams and gives the word representation by summing the representations of the character n-grams [11].

To investigate the effects of unknown words, FastText is added to CONTES as another method, and we have conducted experiments with a subset of the datasets of the BB normalization task.

## Results

### Dimension reduction

**Fig. 1.**
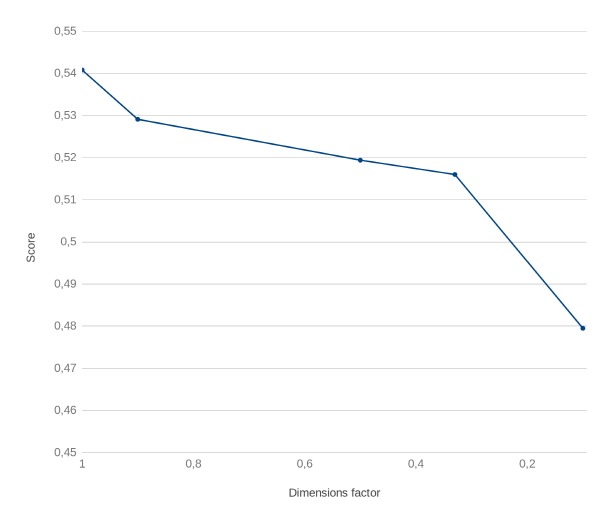
Performance of the method, according to the dimension reduction.

As shown in [7], the PCA and MDS methods had similar behavior, and they both reduced the performance of the CONTES method (Fig. 1). The score is based on the semantic similarity proposed by Wang et al. [12]. The gap is small when the reduction is low, but increases beyond the acceptable performance threshold when it reaches 60% reduction.

The sklearn t-SNE method is not scalable: increasing the reduced space dimension number seems to prevent any execution of reduction. The sklearn description of the method mentions that “It is highly recommended to use another dimensionality reduction method [...] to reduce the number of dimensions to a reasonable amount (e.g., 50), if the number of features is very high” (https://scikit-learn.org/stable/modules/generated/sklearn.manifold.TSNE.html). The initial goal of the method is indeed to enable a 2D/3D representation of a high-dimensional space.

### Overgeneralization

We experimented the aforementioned function for computing SSO by varying the decay factor from zero to one, by steps of 0.1. With a decay factor of one, the function produces the same vectors as the original “Ancestry” method. With a decay factor of zero, the function reproduces a “One Hot” representation.

We measured the impact of the decay factor on the BBs development set, using two metrics. The first metric is the official BBs precision measure that is a mean of the semantic similarity between predicted and reference normalizations, and the semantic similarity rewards predictions that are near the reference. The second metric is a strict measure, where the prediction is only rewarded if it is exactly the same as the reference.

**Fig. 2.**
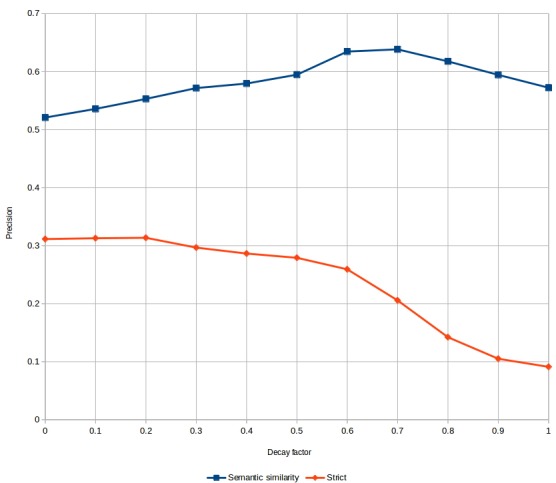
Results with semantic similarity and strict measures.

The results are shown in Fig. 2. The strict measure is necessarily lower, since it does not reward generalization or near-misses. With the semantic similarity measure, we reproduced the results published in Ferre et al.’s study [13] where “Ancestry” (decay = 1) outperforms “One Hot” (decay = 0). We also demonstrated an optimal decay factor between 0.6 and 0.7 that further improves CONTES performance. With the strict measure, we notice that “One Hot” is the best representation.

### Out-of-vocabulary words

Concerning out-of-vocabulary words, our experiments showed no significant difference in performance between FastText and Word2vec.

### Perspectives and conclusion

In this work, we addressed some important issues to normalize biomedical text entities using concepts of ontologies, in the context of small training data. Reduction of dimensions, in the representation of the concepts, appears to be a central issue when trying to support large ontologies. We also tested PCA and MDS methods, and showed that the gap in performance between these methods, and the original CONTES, rapidly widens when the reduction gets high. We also introduced concept embeddings to tackle the dimensional reduction, and we plan to perform follow-up experiments with Node2Vec by more generally considering the other relationships between the concepts in the ontologies. New and large ontologies will be addressed in future work.

By tackling overgeneralization, our results showed a way to enhance CONTES with a function weighing the ontology concepts, based on the depths between the concepts and their ancestors. Out-of-vocabulary words is addressed with FastText, but it does not show significant differences, compared to results without FastText, but we expect that results may change with larger datasets.

The experiments were conducted under specific conditions with reduced datasets, and future work will consider that point.
